# The Campbell Collaboration’s systematic review of school-based anti-bullying interventions does not meet mandatory methodological standards

**DOI:** 10.1186/s13643-022-01998-1

**Published:** 2022-07-18

**Authors:** Julia H. Littell, Dennis M. Gorman

**Affiliations:** 1 Graduate School of Social Work and Social Research, Bryn Mawr College, Bryn Mawr, PA USA; 2 Department of Epidemiology & Biostatistics, School of Public Health, Texas A&M University, College Station, TX USA

**Keywords:** Systematic review, Campbell Collaboration, Risk of bias assessment, Methodological standards, Selective outcome reporting, Outcome reporting bias, Study registration

## Abstract

**Background:**

Many published reviews do not meet the widely accepted PRISMA standards for systematic reviews and meta-analysis. Campbell Collaboration and Cochrane reviews are expected to meet even more rigorous standards, but their adherence to these standards is uneven. For example, a newly updated Campbell systematic review of school-based anti-bullying interventions does not appear to meet many of the Campbell Collaboration’s mandatory methodological standards.

**Issues:**

In this commentary, we document methodological problems in the Campbell Collaboration's new school-based anti-bullying interventions review, including (1) unexplained deviations from the protocol; (2) inadequate documentation of search strategies; (3) inconsistent reports on the number of included studies; (4) undocumented risk of bias ratings; (5) assessments of selective outcome reporting bias that are not transparent, not replicable, and appear to systematically underestimate risk of bias; (6) unreliable assessments of risk of publication bias; (7) use of a composite scale that conflates distinct risks of bias; and (8) failure to consider issues related to the strength of the evidence and risks of bias in interpreting results and drawing conclusions. Readers who are unaware of these problems may place more confidence in this review than is warranted. Campbell Collaboration editors declined to publish our comments and declined to issue a public statement of concern about this review.

**Conclusions:**

Systematic reviews are expected to use transparent methods and follow relevant methodological standards. Readers should be concerned when these expectations are not met, because transparency and rigor enhance the trustworthiness of results and conclusions. In the tradition of Donald T. Campbell, there is need for more public debate about the methods and conclusions of systematic reviews, and greater clarity regarding applications of (and adherence to) published standards for systematic reviews.

Many published systematic reviews are poorly conducted, and many reviews do not follow widely accepted Preferred Reporting Items for Systematic reviews and Meta-Analysis (PRISMA) standards (http://www.prisma-statement.org), although the quality of reporting of biomedical reviews has improved over time [1]. Campbell Collaboration and Cochrane reviews are expected to meet more rigorous standards. The methodological quality and reporting characteristics of Campbell Collaboration reviews have improved over time, but only 17% of a sample of 96 Campbell reviews were assessed as high quality [2].

Campbell Collaboration systematic reviews aim to provide rigorous, transparent, and unbiased assessments of research evidence, so that readers can have confidence in their methods and conclusions. Their guidelines state: “Every Campbell review is required to have clear criteria for eligible research, an explicit and comprehensive search strategy, systematic and replicable coding and analysis of the key features and findings of the studies reviewed, and an integrative summary of those findings” ([3] p. 5). In 2014, the Campbell Collaboration established an explicit set of Methodological Expectations for Campbell Collaboration Intervention Reviews to guide the conduct and reporting of its reviews [2, 4, 5]. Each MECCIR standard is identified by a number, following C for conduct or R for reporting.

Initially, the updated Campbell Collaboration review of school-based anti-bullying interventions [6] caught our attention because the vast majority of its included studies were rated as having low risk of selective outcome reporting (SOR) bias. Under-reporting and selective reporting of outcomes are common [7–11] and preregistration of behavioral intervention trials is uncommon [12–15], so we were curious to know how this review determined whether “outcomes reported in an evaluation study differ from the outcomes of interest proposed originally” ([6] p. 56). Upon further examination, we found that this review did not appear to meet many mandatory MECCIR standards. Five versions of this review were published outside of the Campbell Collaboration [16–20]. Such wide dissemination of results has the potential to influence educational policy and practice, so it is important to understand the review’s methods and conclusions.

Below we raise concerns about methodological qualities of this review and the confidence that readers can place in its results. We describe the Campbell Collaboration’s response to these concerns, and document a difference between their published standards and publication decisions.

## Methodological issues

### Unexplained deviations from the protocol

In our assessment, the new Campbell Collaboration school-based anti-bullying interventions review does not fully “explain and justify any changes from the protocol” (mandatory MECCIR standard R106). Post hoc changes were made in study inclusion criteria, and 13 previously-included studies were excluded as a result [6]. It is not clear why “other quasi-experimental designs” were excluded from the review but “age cohort designs” were retained, as the latter were likely to have different threats to internal validity [21], including history and testing effects, and other uncontrolled differences between groups [6]. To our knowledge, history and testing effects were not assessed in this review. An earlier report indicated that the largest effect sizes were found in age cohort designs [19]. It is possible that post hoc changes in study inclusion criteria affected overall results, but no sensitivity analyses were provided to assess potential impacts of departures from the protocol on overall results (as per MECCIR C13).

The review’s risk of bias (ROB) assessments and moderator analyses also deviated from plans described in the protocol [22] but these changes were not explained (as required by MECCIR R106). There were no plans for ROB assessment in the protocol (original plans for extraction of data on study qualities focused only on overall study designs and attrition). Plans for moderator analysis were not specific, indicating that meta-regression would be used to “investigate independent influences of program components, methodological quality, features of participants, and design features” ([22] p. 12).

### Inadequate documentation of search strategies

We found that search strategies were not reported in sufficient detail for replication (MECCIR C36, R34, R35, R36, R38, R39). Systematic searches were completed in December 2016 [6], more than 4 years prior to publication. Exact search strings, dates, and limits were not provided for specific databases. It is not clear what sources other than ProQuest Dissertations and Theses Solutions were used to search for grey literature [6]. Five studies were added “after searches” were completed ([6] pp. 6, 17), and a sixth study [23] appears without an explanation.

### Inconsistent reports on the number of included studies

The MECCIR requirements state that reviews should fully account for the status of studies (MECCIR C40, C41, C42) and comment on the potential impact of included studies without useable data (MECCIR R89). We found that these steps were not taken in the school-based anti-bullying interventions review.

At one point, the review states that 88 newly identified studies were included ([6] pp. 17, 51); elsewhere it reports that 79 new studies were included ([6] p. 52); but results are shown for only 74 studies ([6] p. 21), with no explanation for missing studies. Portions of the review indicate that 45 RCTs were included ([6] pp. 2, 21), but results are shown for only 41 RCTs ([6] p. 21); again, with no explanation for missing studies.

The review states that total of 141 (old and new) studies were included, then 13 studies were dropped due to post hoc changes in inclusion criteria, bringing the revised total of included studies to 128 ([6] p. 52). In all, 41 studies were excluded for reasons related to study design or incomplete data (in conflict with MECCIR C40) and 100 studies were included in the meta-analysis. References are provided for 116 included studies.

The status of one study [24] is unclear. The reference for this study appears on a list of excluded studies ([6] p. 95) and a similar citation appears in a table of excluded studies ([6] p. 16); but this study is listed as an included study in three places ([6] pp. 26, 75, 101).

### Undocumented ratings of risks of bias

Campbell Collaboration reviews must “present a ‘Risk of Bias’ and/or ‘Study Quality’ table for each included study, with judgments about risks of bias, and explicit supports for these judgments” (MECCIR R72). Campbell reviews are expected to “justify categorical risk of bias/study quality judgments (e.g., high, low, and unclear) with information [taken] directly from the study” (MECCIR C53). But the Campbell school-based anti-bullying interventions review does not provide support for judgments about risk of bias. The review only listed categorical ratings (L for low risk, U for unclear risk, and H for high risk) and provided an overall (study-level) risk of bias score for 90 studies ([6] Appendix B).

Assessment of the inter-rater reliability of risk of bias (ROB) ratings is considered best practice (MECCIR C45, C52). But there was no systematic double coding in this review and there is no information on the reliability of any data extraction or coding tasks ([6] p. 6).

### Assessment of selective outcome reporting (SOR) bias

The review provided SOR bias ratings for only a subsample of included studies: SOR bias ratings are reported for 89 studies on pages 55 and 56 and for 90 studies in Appendix B, but not for all 100 studies included in the meta-analysis or for any of the 41 studies that met initial inclusion criteria but were not included in meta-analysis. Almost all (94%) of the studies that were rated were characterized as having low risk of SOR bias; only two studies were rated as high risk of SOR, and three were rated unclear.

The review did not use an established ROB tool for assessment of SOR bias. According to the published review, “SOR occurs when the outcomes reported in an evaluation study differ from the outcomes of interest proposed originally. For example, if a trial protocol proposed different outcomes than those actually reported in the publication of the trial results” ([6] p. 56). The review defined two levels of SOR bias: a rating of low risk of SOR bias was assigned when “Outcomes proposed are outcomes that are reported” and high risk of SOR bias was identified when “Outcomes proposed are not the outcomes that are reported” ([6] p. 19). The review does not indicate whether protocols for included studies were retrieved or how it was determined which outcomes were “proposed” for each study if prospectively registered protocols were not available. Further, the review provides no documentation of sources consulted or explanations for SOR bias ratings for each study, as required by Campbell’s mandatory MECCIR standard R72.

In the absence of additional support for assessments of SOR bias, we attempted to replicate these ratings, using the review’s criteria and the references it provided. We assumed that randomized controlled trials (RCTs) were more likely than other study designs to have been prospectively registered or have publicly available protocols, so we retrieved documents cited in relation to 42 unique RCTs listed in Tables 9 and 10 of the review [6]. Table 1 (below) shows the SOR ratings the review provided for these 42 RCTs, along with results of our attempt to verify these ratings for the 41 study reports we were able to locate (we could not retrieve one report from a German journal).

**Table 1:** RCTs focused on school-bullying perpetration and/or victimization

Study ^a^	SOR bias rating in the review^b^	RCT report mentions registry or protocol^c^	Our rating using criteria stated in the review^d^
1. **Baldry and Farrington (2004)** [25]	Low	No	Unclear
2. Beran and Shapiro (2005) [26]	Low	No	Unclear
3. *Berry and Hunt (2009)* [27]	*Low*	*No*	Unclear
*4. Bonell et al. (2015)* [28]	*Low*	*Yes*	*Low*
5. Boulton and Flemington (1996) [29]	Low	No	Unclear
**6. Brown et al. (2011)** [30]	Low	No	Unclear
**7. Chaux et al. (2016)** [31]	Low	No	Unclear
**8. Cissner and Ayoub (2014)** [32]	Low	No	Unclear
*9. Connolly et al. (2015)* [33]	Low	No	Unclear
**10. Cross et al. (2011)** [34]	Low	No	Unclear
**11. DeRosier and Marcus (2005)** [35]	Low	No	Unclear
**12. Domino (2013)** [36]	Low	No	Unclear
**13. Espelage et al. (2015)** [37]	Low	No	Unclear
**14. Fekkes et al. (2006)** [38]	Low	No	Unclear
**15. Fekkes et al. (2016)** [39]	Low	No	Unclear
**16. Fonagy et al. (2009)** [40]	Low	No	Unclear
**17. Frey et al. (2005)** [41]	Low	No	Unclear
**18. Garaigordobil and Martínez-Valderrey (2015)** [42]	Low	No	Unclear
19. Holen et al. (2013) [43]	Low	No	Unclear
**20. Hunt (2007)** [44]	Low	No	Unclear/high
**21. Jenson et al. (2013)** [45]	Low	No	Unclear
*22. Ju et al. (2009)* [46]	*Low*	*No*	Unclear/high
**23. Kaljee et al. (2017)** [47]	Unclear	No	Unclear
**24. Kärnä et al. (2011b)** [48]	Low	No	Unclear
**25. Kärnä et al. (2013)** [49]	Low	No	Unclear
*26. Knowler and Frederickson (2013)* [50]	*Low*	*No*	Unclear
27. Krueger (2010) [51]	Low	No	Unclear
28. Li et al. (2011) [52]	Low	No	Unclear
**29. McLaughlin (2009)** [53]	Low	No	Unclear
30. Meyer and Lesch (2000) [54]	Low	No	Unclear
**31. Nocentini and Menesini (2016)** [55]	Low	No	Unclear
32. Ostrov et al. (2015) [56]	Low	No	Unclear/high
**33. Polanin (2015)** [57]	Low	No	Unclear
**34. Rosenbluth et al. (2004)** [58]	Low	No	Unclear
**35. Spröber et al. (2006)** [59]	Low	Unknown^e^	Unknown
36. Stallard et al. (2013) [60]	Low	Yes	High
*37. Topper (2011)* [61]	*Unclear*	No	Unclear
**38. Trip et al. (2015)** [62]	Low	No	Unclear
**39. Tsiantis et al. (2013)** [63]	Low	No	Unclear
40. Waasdorp et al. (2012) [64]	High	No	Unclear
41. Wölfer and Scheithauer (2014) [24]	Low	No	Unclear/high
**42. Yanagida et al. (2019)** [23]	Low	No	Unclear
Summary	39 Low, 1 High, 2 Unclear	2 Yes, 39 No, 1 Unknown	1 Low 1 High, 4 Unclear/high, 35 Unclear, 1 Unknown

^a^Cited in the review [6]. **Bold font** = RCTs in both Tables 9 and 10 (*k* = 26); normal font = RCTs in Table 9 (perpetration) only (*k* = 9); *Italic font* = in Table 10 (victimization) only (*k* = 7)

^b^From Appendix B of the review [6]

^c^Each document was electronically searched for the words “Registry”, “Registered”, “Registration”, and “Protocol”. “No” = this search did not yield a reference to a registry or study protocol; “Yes” = this search did yield a reference to a registry or publicly available study protocol

^d^Criteria provided in the review ([6] p. 19)

^e^This study is in a German journal. It could not be located through Inter-library Loan; therefore, we could not rate the risk of SOR bias for this study

Only two of the 41 reports on RCTs make reference to trial registration or a public study protocol. The details of these studies are important, as they clearly illustrate issues encountered in assessing risk of SOR bias.

An RCT by Stallard and colleagues [60] was prospectively registered in 2007 [65]. The intervention tested in this trial was not an anti-bullying program (it aimed to prevent depression) and the trial registration record does not mention any intended outcomes related to bullying [65]. Enrollment began in 2008 and data collection began in 2009 [65]. A second protocol for this study, published retrospectively, mentions bullying as one of several secondary outcomes, but does not indicate how bullying outcomes were measured [66]. A third “protocol” for this study appears as an appendix to the 2013 study report and it states that, “The two global items [of the Olweus Bully/Victim Questionnaire] assessing the frequency of self-reported bullying and being the victim of bullying will be used” ([60] p. 105). However, Stallard and colleagues reported results for only one of these two bullying outcomes (perpetration, not victimization) [60]. This study was rated as low risk of SOR in the review, although it meets the review’s criteria for high risk, because bullying outcomes were not mentioned in the initial proposal.

A trial reported by Bonell and colleagues [28] was prospectively registered in 2011 [67], listing aggressive behaviors and bullying as primary outcomes to be measured by the Gatehouse Bullying Scale (12 items) and the Ayan Aba Youth Project subscale on aggressive behavior (4 items). Results are reported for all 16 items at baseline and follow-up [28]. The study was rated as low risk of SOR bias in the review [6], which is justified based on a comparison of the trial registry record and the study report.

The remaining 39 reports on RCTs included no references to trial registration or public protocols (see Table 1). The review rated 36 of these trials as low risk of SOR bias, two as unclear, and one as high risk. Using the review’s stated criteria, we rated all 39 of these RCTs as having unclear risk of SOR bias; because no prospectively registered protocol was cited in these trials, there was no way to determine which outcomes were initially proposed.

A closer look at the trial reports raised additional questions about the review’s assessment of SOR bias. Two studies collected data on the Olweus bully/victim questionnaire but reported results for only one of the two outcomes assessed with this instrument (one study reported perpetration only [24], another reported victimization only [46]). A third study obtained outcome measures on four types of bullying (proactive physical bullying, reactive physical bullying, proactive relational bullying, and reactive relational bullying), but collapsed proactive and reactive measures in the reported analysis ([56] p. 450). A fourth trial obtained data on two of the Attitude to Bullying subscales but included only one of these subscales in the published analysis ([44] p. 23). We coded these four studies as having Unclear/High risks of SOR. The review rated a fifth study [64] as having a high risk of SOR bias; given the lack of a study protocol or trial registration record, we rated the risk of SOR in this study as unclear.

In summary, using the review’s criteria for SOR bias, we rated 35 of 41 trials as having unclear risk, four as unclear/high risk, one trial as high risk, and one trial as low risk (see Table 1). As shown in Table 2, our ratings agree with those of the review on only 3 of 41 trials (proportion of agreement= 7%, Cohen’s kappa = 0.003), a very low level of agreement.

**Table 2:** Reliability of ratings of SOR bias for 41 RCTs^a^

	Our SOR ratings based on criteria stated in the review ([6] p. 19)		
SOR ratings in the review ([6] Appendix B)	Low risk	Unclear or unclear/high	High risk
Low risk	1	36	1
Unclear	0	2	0
High risk	0	1	0

^a^For 41 trials with two sets of ratings: proportion of agreement = 7% (3/41); Cohen’s kappa = 0.003

The review rated all but two (45) non-RCTs as having low risk of SOR bias. We did not attempt to verify these ratings because we did not expect to find protocols for these studies.

Selective reporting of outcomes is a pervasive problem in evaluations of interventions in the social and health sciences [8, 9, 11], and SOR bias remains a clear threat to the validity of systematic reviews [7, 68]. We found that the methods used in this review to assess SOR are not transparent, not replicable, and appear to systematically underestimate risk of SOR bias in the included studies.

### Unreliable assessments of risk of publication bias

To assess the risk of publication bias, the review relied on (a) visual inspection of funnel plots and (b) trim and fill analysis. Empirical studies show that visual assessment of funnel plot asymmetry is unreliable [69, 70]. It is not clear why reviewers did not use Egger’s test or another statistical test of funnel plot asymmetry. Trim and fill analysis is not reliable in the presence of between-study heterogeneity [71, 72], and substantial heterogeneity is apparent in this review ([6] p. 76). Results of trim and fill analysis depend heavily on which estimators are used [73], but estimators were not specified in the review. In sum, the review does not provide convincing evidence for its conclusion that publication bias was unlikely or “not present” in its meta-analyses ([6] pp. 2, 74, 76).

### Use of a composite scale that conflates distinct risks of bias

The review states, “Scores on each of the risk of bias items were summed to estimate a total risk of bias score. This continuous variable was then used to examine the relationship between intervention effectiveness and risk of bias in meta-regression models” ([6] p. 76). This is at odds with a mandatory MECCIR standard (C51), which states that “Campbell reviews should not use composite scales, indices, or other measures that conflate multiple measures of risk of bias/study quality into a single score (e.g., using an average scale that combines measures of allocation concealment, attrition, and baseline equivalence measures). These composite quality scales can be misleading and should not be used in a Campbell Collaboration review. Instead, any risk of bias/study quality coding should isolate unique measures of quality (e.g., separate measures for allocation concealment, attrition, spillover, selective outcome reporting, selective analysis reporting, and baseline equivalence)” ([4] p. 16).

### Considering the strength of the evidence in interpreting results and drawing conclusions

The review rated 30% to 40% of (*k* = 89 to 91) included studies as having high risks of bias on: allocation sequence, allocation concealment, contamination, and conflicts of interest ([6] pp. 55–56, Appendix B). It reported a mismatch between units of allocation and units of analysis in most studies, noting that this was “a threat” to the findings ([6] p. 86). Studies rated high risk of conflict of interest (COI) had significantly larger effect sizes than studies rated low risk of COI ([6] p. 84). Yet there was no discussion of issues of risk of bias (or study quality) in the review’s abstract (mandatory MECCIR items R11 and R12) or in the discussion of limitations of the review (MECCIR mandatory item R100). We think these issues should have been presented as caveats for readers to consider when evaluating the review’s conclusions that school-based anti-bullying programs “are effective” and their “effect sizes are modest.”

## Conclusions

The new (updated) Campbell Collaboration systematic review of school-based anti-bullying interventions [6] does not appear to meet many of the Campbell Collaboration’s mandatory MECCIR standards (e.g., C13, C20, C22, C36, C51, R11, R12, R34, R35, R36, R38, R72, R89, R100, R106). These standards were created to support the conduct and reporting of systematic reviews. In our assessment, the review’s deviations from mandatory MECCIR standards mean that its searches are not replicable, inclusion decisions are not transparent (numbers of included studies fluctuation for reasons that are unclear), and bias assessments are not supported with evidence. Most striking, our assessment of SOR bias showed very little agreement with the review’s SOR bias ratings, even though the same criteria were used in both assessments. We believe that this review underestimates risks of SOR and publication bias, which may lead readers to think that the evidence base for this review is more complete and more trustworthy than it really is. Further, the review does not fully consider issues related to the strength of the evidence and risks of bias when presenting its conclusions. We believe this raises questions about the confidence readers can place in this review.

Our assessment also raises concerns about the editorial process that led to publication of this review. The Campbell Collaboration MECCIR reporting standards state that “a new review will not be published if [a mandatory] standard is not met” ([5] p. 3). Give our assessment that these standards were not met, it is not clear to us why this review was published in *Campbell Systematic Reviews*.

Clear standards for the conduct and reporting of systematic reviews provide important guidance for reviewers and editors. Readers should be able to assume that established guidelines were followed, and mandatory requirements were met. This is especially important if readers are using systematic reviews to guide their decisions as to which intervention programs to implement. In a world of diminishing resources for social interventions, there are opportunity costs associated with selecting poorly evaluated interventions that produce unreliable, biased, or false positive results. Systematic reviews are intended to differentiate weak studies from rigorous evaluations that produce valid results, using thorough assessments of common sources of bias, such as SOR. Our confidence in this review was reduced by the lack of transparency both within the review and in the editorial process.

With regard to the latter, we should note that Campbell Collaboration editors declined to publish our comments on this review (they would only consider publishing a brief comment on one issue: assessment of SOR bias). They also declined to publish a statement alerting readers to the fact that concerns had been raised about whether the updated school-based anti-bullying interventions review met many of the mandatory MECCIR standards.

We think that greater transparency about the application of published standards (including questions about whether a specific review has met these standards) and about editorial and publication decisions is needed to instill readers’ confidence in these processes and improve the quality of future systematic reviews. If mandatory MECCIR standards are not followed in published Campbell reviews, then there is a real gap between the Campbell Collaboration’s public criteria and its editorial and publication decisions. This gap is not transparent and the de facto standards for Campbell Collaboration systematic reviews are not clear to us. Lack of transparency and erosion of published standards may diminish the rigor of research reviews and undermine public confidence in them.

For us this raises questions about whether the Campbell Collaboration is living up to the legacy of Donald T. Campbell, the US social scientist for whom the Collaboration was named. In 1986, Campbell wrote, “Science requires a disputatious community of ‘truth seekers’” ([74] p. 35). He added, “The norms of science are explicitly anti-authoritarian [and] antitraditional…. The community of scientists is to stay together in focused disputation, attending to each other’s arguments and illustrations, mutually monitoring and ‘keeping each other honest’…” ([74] p. 35). Following Donald T. Campbell, we believe there is ongoing need for more public debate about the methods and conclusions of systematic reviews and all forms of empirical research. Further, editorial and publication decisions should be more transparent and open to public debate. Science cannot flourish otherwise.

## Data Availability

All data generated during this study are included in this article.

## Abbreviations

COI: Conflict of interest
MECCIR: Methodological Expectations of Campbell Collaboration Intervention Reviews
PRISMA: Primary Reporting Items for Systematic reviews and Meta-Analysis
RCT: Randomized controlled trial
ROB: Risk of bias
SOR: Selective outcome reporting

## References

[1] Page MJ, Shamseer L, Altman DG, Tetzlaff J, Sampson M, Tricco AC (2016). Epidemiology and reporting characteristics of systematic reviews of biomedical research: a cross-sectional study. Low N, editor. PLoS Med.

[2] Wang X, Welch V, Li M, Yao L, Littell J, Li H (2021). The methodological and reporting characteristics of Campbell reviews: a systematic review. Campbell Syst Rev.

[3] The Campbell Collaboration (2020). Campbell systematic reviews: policies and guidelines, Version 1.7, page 5.

[4] The Methods Coordinating Group of the Campbell Collaboration. Methodological expectations of Campbell Collaboration intervention reviews: conduct standards. The Campbell Collaboration; 2019 Oct. Available from: https://campbellcollaboration.org/library/campbell-methods-conduct-standards.html. Accessed 10 Mar 2022.

[5] The Methods Coordinating Group of the Campbell Collaboration. Methodological expectations of Campbell Collaboration intervention reviews: reporting standards. The Campbell Collaboration; 2019 Oct. Available from: https://campbellcollaboration.org/library/campbell-methods-reporting-standards.html. Accessed 10 Mar 2022.

[6] Gaffney H, Ttofi MM, Farrington DP. Effectiveness of school-based programs to reduce bullying perpetration and victimization: an updated systematic review and meta-analysis. Campbell Syst Rev. 2021;17(2). 10.1002/cl2.1143. 10.1002/cl2.1143 PMC8356322 37131921

[7] Kirkham JJ, Dwan KM, Blümle A, von Elm E, Williamson PR (2016). How much participant outcome data Is missing from sight: Findings from a cohort of trials submitted to a German research ethics committee. PLoS One.

[8] Pigott TD, Valentine JC, Polanin JR, Williams RT, Canada DD (2013). Outcome-reporting bias in education research. Educ Res.

[9] Dwan K, Gamble C, Williamson PR, Kirkham JJ, the Reporting Bias Group (2013). Systematic review of the empirical evidence of study publication bias and outcome reporting bias — an updated review. PLoS One.

[10] Schriger DL, Savage DF, Altman DG. Presentation of continuous outcomes in randomised trials: an observational study. BMJ. 2012;345(Dec18 2):e8486. 10.1136/bmj.e8486 PMC3668620 23249670

[11] Song F, Parekh S, Hooper L, Loke Y, Ryder J, Sutton A, et al. Dissemination and publication of research findings: an updated review of related biases. Health Technol Assess. 2010;14(8) Available from: https://www.journalslibrary.nihr.ac.uk/hta/hta14080/. Accessed 11 Mar 2022. 10.3310/hta14080 20181324

[12] Taylor NJ, Gorman DM (2022). Registration and primary outcome reporting in behavioral health trials. BMC Med Res Methodol.

[13] Bradley HA, Rucklidge JJ, Mulder RT (2017). A systematic review of trial registration and selective outcome reporting in psychotherapy randomized controlled trials. Acta Psychiatr Scand.

[14] Azar M, Riehm KE, McKay D, Thombs BD (2015). Transparency of outcome reporting and trial registration of randomized controlled trials published in the journal of consulting and clinical psychology. Schooling CM, editor. PLoS One.

[15] Milette K, Roseman M, Thombs BD (2011). Transparency of outcome reporting and trial registration of randomized controlled trials in top psychosomatic and behavioral health journals: A systematic review. J Psychosom Res.

[16] Gaffney H, Farrington DP, White H (2021). Anti-bullying programmes: Toolkit technical report.

[17] Gaffney H, Farrington DP, Espelage DL, Ttofi MM (2019). Are cyberbullying intervention and prevention programs effective? A systematic and meta-analytical review. Aggress Violent Behav.

[18] Gaffney H, Farrington DP, Ttofi MM (2019). Examining the Effectiveness of School-Bullying Intervention Programs Globally: a Meta-analysis. Int J Bullying Prev.

[19] Gaffney H, Ttofi MM, Farrington DP (2019). Evaluating the effectiveness of school-bullying prevention programs: An updated meta-analytical review. Aggress Violent Behav.

[20] Gaffney H, Ttofi MM, Farrington DP (2021). What works in anti-bullying programs? Analysis of effective intervention components. J Sch Psychol.

[21] Farrington DP, Ttofi MM. School-based programs to reduce bullying and victimization. Campbell Syst Rev. 2009;5(1). 10.4073/csr.2009.6. 10.1002/cl2.1143 PMC8356322 37131921

[22] Farrington DP, Baldry AC, Kyvsgaard B, Ttofi MM (2008). PROTOCOL: effectiveness of programs to prevent school bullying. Campbell Syst Rev.

[23] Yanagida T, Strohmeier D, Spiel C (2019). Dynamic change of aggressive behavior and victimization among adolescents: effectiveness of the ViSC program. J Clin Child Adolesc Psychol.

[24] Wölfer R, Scheithauer H (2014). Social influence and bullying behavior: intervention-based network dynamics of the fairplayer.manual bullying prevention program: Social Influence and Bullying. Aggress Behav.

[25] Baldry AC, Farrington DP. Evaluation of an intervention program for the reduction of bullying and victimization in schools. Aggr Behav. 2004;30(1):1–15.

[26] Beran T, Shapiro B. Evaluation of an anti-bullying program: student reports of knowledge and confidence to manage bullying. Can J Educ / Revue canadienne de l’éducation. 2005;28(4):700.

[27] Berry K, Hunt CJ. Evaluation of an intervention program for anxious adolescent boys who are bullied at school. J Adolesc Health. 2009;45(4):376–82. 10.1016/j.jadohealth.2009.04.023 19766942

[28] Bonell C, Fletcher A, Fitzgerald-Yau N, Hale D, Allen E, Elbourne D (2015). Initiating change locally in bullying and aggression through the school environment (INCLUSIVE): a pilot randomised controlled trial. Health Technol Assess.

[29] Boulton MJ, Flemington I. The effects of a short video intervention on secondary school pupils’ involvement in definitions of and attitudes towards bullying. School Psychol Int. 1996;17(4):331–45.

[30] Brown EC, Low S, Smith BH, Haggerty KP. Outcomes from a school-randomized controlled trial of steps to respect: a bullying prevention program. 2011;40(3):423–43.

[31] Chaux E, Velásquez AM, Schultze-Krumbholz A, Scheithauer H. Effects of the cyberbullying prevention program media heroes (Medienhelden) on traditional bullying: effects of media heroes on traditional bullying. Aggr Behav. 2016;42(2):157–65. 10.1002/ab.21637 26879895

[32] Cissner AB, Ayoub LH. Building healthy teen relationships: an evaluation of the fourth R curriculum with middle school students in the Bronx. USA: Center for Court Innovation; 2014.

[33] Connolly J, Josephson W, Schnoll J, Simkins-Strong E, Pepler D, MacPherson A, et al. Evaluation of a youth-led program for preventing bullying, sexual harassment, and dating aggression in middle schools. J Early Adolesc. 2015;35(3):403–34.

[34] Cross D, Monks H, Hall M, Shaw T, Pintabona Y, Erceg E, et al. Three‐year results of the Friendly Schools whole-of-school intervention on children’s bullying behaviour. Br Educ Res J. 2011;37(1):105–29.

[35] DeRosier ME, Marcus SR. Building friendships and combating bullying: effectiveness of S.S.GRIN at one-year follow-up. J Clin Child Adolesc Psychol. 2005;34(1):140–50. 10.1207/s15374424jccp3401_13 15677288

[36] Domino M. Measuring the impact of an alternative approach to school bullying. J School Health. 2013;83(6):430–7. 10.1111/josh.12047 23586888

[37] Espelage DL, Rose CA, Polanin JR. Social-emotional learning program to reduce bullying, fighting, and victimization among middle school students with disabilities. Remedial Spec Educ. 2015;36(5):299–311.

[38] Fekkes M, Pijpers FIM, Verloove-Vanhorick SP. Effects of antibullying school program on bullying and health complaints. Arch Pediatr Adolesc Med. 2006;160(6):638. 10.1001/archpedi.160.6.638 16754827

[39] Fekkes M, van de Sande MCE, Gravesteijn JC, Pannebakker FD, Buijs GJ, Diekstra RFW, et al. Effects of the Dutch Skills for Life program on the health behavior, bullying, and suicidal ideation of secondary school students. Health Educ. 2016;116(1):2–15.

[40] Fonagy P, Twemlow SW, Vernberg EM, Nelson JM, Dill EJ, Little TD, et al. A cluster randomized controlled trial of child-focused psychiatric consultation and a school systems-focused intervention to reduce aggression. J Child Psychol Psychiatry. 2009;50(5):607–16. 10.1111/j.1469-7610.2008.02025.x 19207633

[41] Frey KS, Hirschstein MK, Snell JL, Edstrom LVS, MacKenzie EP, Broderick CJ. Reducing playground bullying and supporting beliefs: an experimental trial of the steps to respect program. Dev Psychol. 2005;41(3):479–90. 10.1037/0012-1649.41.3.479 15910156

[42] Garaigordobil M, Martínez-Valderrey V. Effects of Cyberprogram 2.0 on “face-to-face” bullying, cyberbullying, and empathy. Psicothema. 2015;(27.1):45–51. 10.7334/psicothema2014.78 25633769

[43] Holen S, Waaktaar T, Lervåg A, Ystgaard M. Implementing a universal stress management program for young school children: are there classroom climate or academic effects? Scand J Educ Res. 2013;57(4):420–44.

[44] Hunt C (2007). The Effect of an Education Program on Attitudes and Beliefs about Bullying and Bullying Behaviour in Junior Secondary School Students. Child Adolesc Ment Health.

[45] Jenson JM, Brisson D, Bender KA, Williford AP. Effects of the youth matters prevention program on patterns of bullying and victimization in elementary and middle school. Soc Work Res. 2013;37(4):361–72.

[46] Ju Y, Wang S, Zhang W (2009). Intervention research on school bullying in primary schools. Front Educ China.

[47] Kaljee L, Zhang L, Langhaug L, Munjile K, Tembo S, Menon A, et al. A randomized-control trial for the teachers’ diploma programme on psychosocial care, support and protection in Zambian government primary schools. Psychol Health Med. 2017;22(4):381–92. 10.1080/13548506.2016.1153682 26965476

[48] Kärnä A, Voeten M, Little TD, Poskiparta E, Kaljonen A, Salmivalli C. A large-scale evaluation of the KiVa antibullying program: grades 4-6: evaluation of KiVa antibullying program. Child Dev. 2011b;82(1):311–30. 10.1111/j.1467-8624.2010.01557.x 21291444

[49] Kärnä A, Voeten M, Little TD, Alanen E, Poskiparta E, Salmivalli C. Effectiveness of the KiVa Antibullying Program: Grades 1–3 and 7–9. J Educ Psychol. 2013;105(2):535–51.

[50] Knowler C, Frederickson N. Effects of an emotional literacy intervention for students identified with bullying behaviour. Educ Psychol. 2013;33(7):862–83. 10.1080/01443410.2013.785052 PMC4579054 26494932

[51] Krueger LM. The implementation of an anti-bullying program to reduce bullying behaviors on elementary school buses [Internet] [Ed.D.]. ProQuest Dissertations and Theses. Ann Arbor: D’Youville College; 2010.

[52] Li KK, Washburn I, DuBois DL, Vuchinich S, Ji P, Brechling V, et al. Effects of the Positive Action programme on problem behaviours in elementary school students: A matched-pair randomised control trial in Chicago. Psychol Health. 2011;26(2):187–204. 10.1080/08870446.2011.531574 21318929

[53] McLaughlin LP. The effect of cognitive behavioral therapy and cognitive behavioral therapy plus media on the reduction of bullying and victimization and the increase of empathy and bystander response in a bully prevention program for urban sixth -grade students [Internet] [Ph.D.]. ProQuest Dissertations and Theses. Ann Arbor: The University of Toledo; 2009.

[54] Meyer N, Lesch E. An analysis of the limitations of a behavioural programme for bullying boys from a subeconomic environment. null. 2000;12(1):59–69.

[55] Nocentini A, Menesini E. KiVa anti-bullying program in Italy: evidence of effectiveness in a randomized control trial. Prev Sci. 2016;17(8):1012–23. 10.1007/s11121-016-0690-z 27488457

[56] Ostrov JM, Godleski SA, Kamper-DeMarco KE, Blakely-McClure SJ, Celenza L (2015). Replication and extension of the early childhood friendship project: effects on physical and relational bullying. Stormont M, editor. School Psychol Rev.

[57] Polanin MK. [Ph.D.]. ProQuest Dissertations and Theses. [Ann Arbor]: Loyola University Chicago; 2014. Effects of cultural awareness training in conjunction with an established bullying prevention program [Internet].

[58] Rosenbluth B, Whitaker DJ, Sanchez E, Valle LA. The Expect Respect project: preventing bullying and sexual harassment in US elementary schools. In: Pepler D, Rigby K, Smith PK, editors. Bullying in Schools: How Successful Can Interventions Be? Cambridge: Cambridge University Press; 2004. p. 211–34.

[59] Spröber N, Schlottke PF, Hautzinger M. ProACT + E: Ein Programm zur Prävention von “bullying“ an Schulen und zur Förderung der positiven Entwicklung von Schülern. Zeitschrift für Klinische Psychologie und Psychotherapie. 2006;35(2):140–50.

[60] Stallard P, Phillips R, Montgomery A, Spears M, Anderson R, Taylor J, et al. A cluster randomised controlled trial to determine the clinical effectiveness and cost-effectiveness of classroom-based cognitive–behavioural therapy (CBT) in reducing symptoms of depression in high-risk adolescents. Health Technol Assess. 2013;17(47). 10.3310/hta17470. 10.3310/hta17470 PMC4781207 24172024

[61] Topper L. Bullying victimisation and alcohol-misuse in adolescence: Investigating the functional relationship and new prevention strategies [Doctoral dissertation]. King’s College London (University of London); 2012.

[62] Trip S, Bora C, Sipos-Gug S, Tocai I, Gradinger P, Yanagida T, et al. Bullying prevention in schools by targeting cognitions, emotions, and behavior: Evaluating the effectiveness of the REBE-ViSC program. J Couns Psychol. 2015;62(4):732–40. 10.1037/cou0000084 26376177

[63] Tsiantis ACJ, Beratis IN, Syngelaki EM, Stefanakou A, Asimopoulos C, Sideridis GD, et al. The effects of a clinical prevention program on bullying, victimization, and attitudes toward school of elementary school students. Behav Disord. 2013;38(4):243–57.

[64] Waasdorp TE. The impact of schoolwide positive behavioral interventions and supports on bullying and peer rejection: a randomized controlled effectiveness trial. Arch Pediatr Adolesc Med. 2012;166(2):149. 10.1001/archpediatrics.2011.755 22312173

[65] Stallard P. A single blind randomised controlled trial to determine the effectiveness of group Cognitive Behaviour Therapy (CBT) in the prevention of depression in high risk adolescents. ISRCTN. Available from: http://www.isrctn.com/ISRCTN19083628. Accessed 10 Mar 2022.

[66] Stallard P, Montgomery AA, Araya R, Anderson R, Lewis G, Sayal K (2010). Protocol for a randomised controlled trial of a school based cognitive behaviour therapy (CBT) intervention to prevent depression in high risk adolescents (PROMISE). Trials..

[67] Viner R (2011). INitiating Change Locally in bUllyIng and aggression through the School EnVironment. ISRCTN.

[68] Norris SL, Holmer HK, Ogden LA, Fu R, Abou-Setta AM, Viswanathan MS, et al. Selective outcome reporting as a source of bias in reviews of comparative effectiveness. Rockville, MD: Agency for Healtcare Research and Quality; 2012 Aug. Report No.: AHRQ Publication No. 12-EHC110-EF. Available from: www.effectivehealthcare.ahrq.gov/reports/final.cfm. Accessed 11 Mar 2022. 22993870

[69] Lau J, Ioannidis JPA, Terrin N, Schmid CH, Olkin I (2006). The case of the misleading funnel plot. BMJ..

[70] Terrin N, Schmid CH, Lau J (2005). In an empirical evaluation of the funnel plot, researchers could not visually identify publication bias. J Clin Epidemiol.

[71] Peters JL, Sutton AJ, Jones DR, Abrams KR, Rushton L, Moreno SG (2010). Assessing publication bias in meta-analyses in the presence of between-study heterogeneity. J R Stat Soc A Stat Soc.

[72] Terrin N, Schmid CH, Lau J, Olkin I (2003). Adjusting for publication bias in the presence of heterogeneity. Stat Med.

[73] Shi L, Lin L. The trim-and-fill method for publication bias: practical guidelines and recommendations based on a large database of meta-analyses. Medicine. 2019;98(23) Available from: https://journals.lww.com/md-journal/Fulltext/2019/06070/The_trim_and_fill_method_for_publication_bias_.70.aspx. 10.1097/MD.0000000000015987 PMC6571372 31169736

[74] Campbell DT, Dunn WN (1998). The experimenting society. The experimenting society: essays in honor of Donald T Campbell.

